# All-carbon based graphene field effect transistor with graphitic electrodes fabricated by e-beam direct writing on PMMA

**DOI:** 10.1038/srep12198

**Published:** 2015-07-21

**Authors:** Wei Chen, Yayun Yu, Xiaoming Zheng, Shiqiao Qin, Fei Wang, Jingyue Fang, Guang Wang, Chaocheng Wang, Li Wang, Gang Peng, Xue-Ao Zhang

**Affiliations:** 1 College of Science, National University of Defense Technology, Changsha 410073, P. R. China; 2 State Key Laboratory of high Performance Computing, National University of Defense Technology, Changsha 410073, P. R. China; 3 Department of Physics, Nanchang University, Nanchang 330031, P. R. China

## Abstract

A so called all-carbon based graphene field effect transistor (GFET) in which the electrodes are composed of graphite-like nano-sheets instead of metals in the traditional devices is fabricated by one-step e-beam direct writing (EBDW). It is also found that the graphite-like nano-sheets in electrodes are perpendicular to the channel graphene, which is confirmed by the transmission electron microscopy (HRTEM). The one-step fabrication of the carbonaceous electrodes is more convenient and lower-cost comparing to the preparation of traditional metal electrodes and can be applied to many other nano-electronic devices.

Graphene, a two-dimensional carbon material, has attracted wide attentions from both condensed-matter physicists and chip-makers for almost a decade^1^,^2^,^3^,^4^,^5^. Graphene field effect transistors (GFETs) have developed rapidly since it is considered as an option for post-silicon electronics^6^. In traditional GEFT devices, metal/graphene contacts for source and drain electrodes are inevitable, however, such metal/graphene contacts could seriously hinder the performances of graphene transistors, especially the high-speed traveling of the carriers^7^,^8^,^9^,^10^,^11^. The band-structure of graphene would be significantly altered by chemisorption on Co, Ni, and Pd. In addition, the Fermi energy in graphene can be moved away from the conical points even by weak adsorption of Al, Cu, Ag, Au, and Pt, which results in the doping of graphene with either electrons or holes^12^. The high-resolution angle-resolved photoemission studies show that substantial band gap opens in proportion to the doping effect at Ag and Cu/graphene contacts^13^. Especially, the modification of metal on the electronic structure of graphene not only occurs in the area underneath the metal electrodes but also affects the adjacent graphene regions hundreds nanometers away from the electrodes^14^,^15^. Besides the electronic structure, the electrical transport in graphene would also be impacted by the metal/graphene contacts. The electron-hole asymmetry and sublinear conductance as a function of carrier density mainly originate from the pinning of the charge density below the metal especially in short channel devices^16^,^17^. Therefore, a proper material for electrodes is very important for the realistic application of high-performance graphene-based devices. Carbonaceous electrode is a potential proposal for high-performance graphene-based electronics. Several groups have already made efforts towards the all-carbon electronics. Yang *et al.*^18^ reported a fabrication of all-graphene devices via layer-by-layer thinning of graphene by hydrogen-plasma etching. Wang *et al.*^19^ created all-carbon electrodes by ink jet printing with solution-processable graphene oxide sheets.

In this study, an all-carbon based GFET in which the electrodes are composed of graphite nano-sheets instead of metals has been fabricated by one-step e-beam direct writing (EBDW) on poly(methyl methacrylate) (PMMA) which is the most widely used polymeric electron resist due to its high resolution, stability, and compatibility with other processing steps^20^,^21^. Although PMMA is well known as a positive e-beam resist, it can be transformed to a high-resolution (sub-10-nm) negative resist if the e-beam irradiation is high enough (overexposure)^22^,^23^,^24^,^25^,^26^,^27^,^28^. It is found that the negative PMMA can be graphitized by post-annealing treatment. Structural analysis including Raman scattering and high-resolution transmission electron microscopy (HRTEM) reveal that these graphitized and conductive electrodes are composed by graphite-like nano-sheets perpendicular to the channel graphene. The electrical measurements unambiguously demonstrated that the performances of such all-carbon based GFET are comparable to those of the devices with the metallic electrodes. The negative PMMA with good electrical conductivity and high-resolution is promising for the fabrication of nano-electronic devices and provides the possibility to fabricate all carbon-based GFET devices.

## Results and Discussion

**Figure 1:**
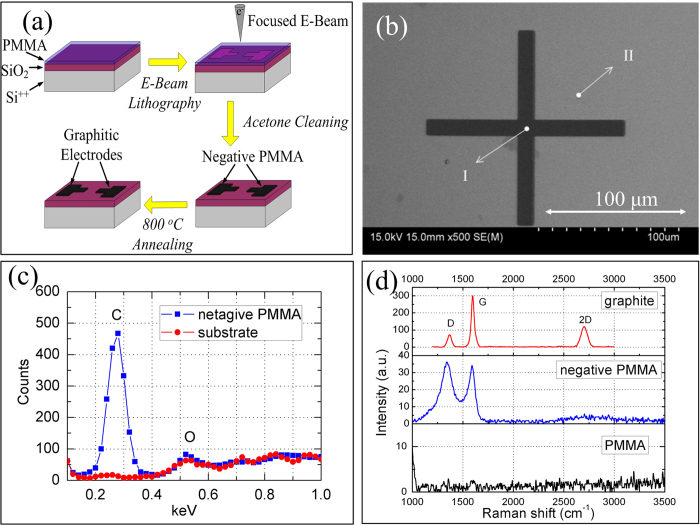
(**a**) The technological process of fabrication of the carbonaceous electrodes. (**b**) A cross bar of the negative PMMA prepared on a silicon substrate following the process in (**a**) for characterization. (**c**) The contradistinction of EDS spectrum between the area I (center of the cross pattern) and the area II (substrate) in (**b**). (**d**) The contradistinction of Raman spectrum between crystal graphite and the negative PMMA. The wavelength of the incident laser was 532 nm.

The technological process of the carbonaceous electrodes is illustrated in Fig. 1(a) and the details are described in the *Methods*. The resistivity of the carbonaceous electrode is measured to be about 4.8 × 10^−3^ Ω · cm [Supporting Information], which is much smaller than that of a heavily doped silicon (~10^−2^ Ω · cm^29^) and actually comparable to the in-plane resistivity for graphite(2.5–5.0 × 10^−4^ Ω · cm^30^). Therefore, such film could be good enough to act as electrodes in electronic devices.

The chemical components and structure of the annealed negative PMMA film were studied to understand the conductive mechanism. To avoid the influence of the substrate, a cross bar of the negative PMMA film was prepared on a silicon substrate, as shown in Fig. 1(b). The energy dispersive spectrometer (EDS) spectra were taken at the center of the cross bar (I) (marked by rectangles in Fig. 1(c)) and the substrate Comparing the EDS spectrum of the negative PMMA with that of the substrate, it is obvious that the negative PMMA is a carbonaceous material indeed and the oxygen atoms are almost negligible. The disappearance of oxygen atoms is caused by the volatile groups such CO_2_, H_2_ and CH_3_O via e-beam and annealing induced decomposition of the PMMA^21^,^28^. Figure 1(d) shows the comparison of the Raman spectrum between the bulk graphite and the negative PMMA in the Fig. 1(b). There are two obvious peaks at about 1350 and 1580 cm^−1^, which correspond to the D and G peaks of graphite, respectively. The G peak signifies the mono- or poly-crystalline graphite and the D peak reflects the defects and disordering of the graphite^31^,^32^,^33^,^34^,^35^. Although the broad 2D peak around 2700 cm^−1^ at the spectrum of the negative PMMA is not clear as that in the spectrum for the bulk graphite, the presence of such 2D peak corresponds to the other nano-graphites^35^,^36^,^37^. Therefore, the Raman measurements indicate that the prepared negative PMMA is indeedly well graphitized.

**Figure 2:**
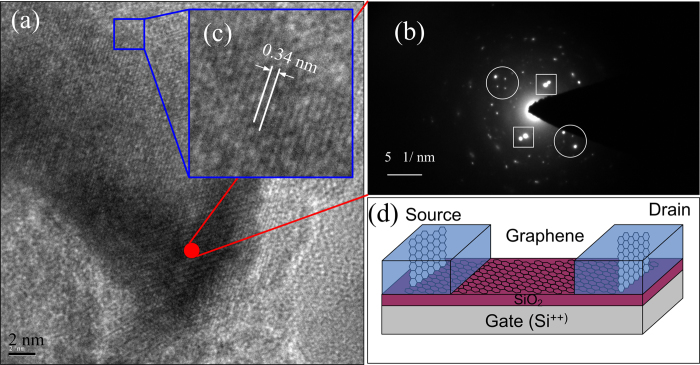
Structure of the carbonaceous electrode. (**a**) The HRTEM picture of the negative PMMA prepared on a Cu grid, in which the zoom-in part (**b**) clearly shows the straight fringes with the distance of about 0.34 nm. (**c**) The SAED pattern at the area of the marked red point in (**a**). (**d**) The diagrammatic sketch of the all-carbon based GFET indicating the vertical contacts between the channel graphene and the graphite sheets in the electrodes.

To study the detailed structure, a high-resolution transmission electron microscopy (HRTEM) was employed to analyze the negative PMMA. The sample for the HRTEM was directly prepared on a Cu grid via the same processes and conditions in the Fig. 1(a). Figure 2(a) gives a high-resolution transmission electron microscopy (HRTEM) image for the negative PMMA prepared via the same processes and conditions. The crystallization of the negative PMMA film is firstly confirmed by the selected area electron diffraction (SAED) pattern, as shown in the Fig. 2(b). From the SAED pattern, several couples of symmetrical primary diffractive spots (as marked by the circle and square boxes) and lots of random high-order diffractive spots can be seen, which indicates that the fragment is really polycrystalline. The HRETM image demonstrates that the negative PMMA consists of some crystallized nano-fragments with different crystal orientations. In addition, the embedded nano-crystals are a kind of layered structure with the spacing of ~0.34 nm, as shown by the partial zoom-in picture (Fig. 2(c)), which matches well with the layered structure of graphite^30^. Combination of HRTEM and Raman measurements strongly indicate that the crystallized nano-fragments in the negative PMMA are graphite nano-sheets. It is the formation of nano-fragmental and the random arrangements of the graphite sheets that cause the intense D peak and the wide 2D peak in the Raman spectrum of the negative PMMA.

The graphitization of the negative PMMA may originate from two concurrent processes: the lost of oxygen and hydrogen atoms (i.e. the deoxygenation and dehydrogenation) and the self-assembling of carbon-carbon (C-C) covalent bonds. Hydrocarbon gas can be decomposed into amorphous carbon by losing oxygen and hydrogen atoms under high-intensity electron irradiation in electron-beam-induced deposition (EBID) process^38^,^39^. Since PMMA is a kind of polymer, the macromolecular chains in PMMA would be broken into small molecular chains under electron irradiation. As long as the irradiation dose is large enough (i.e. overexposure of positive PMMA), most of the oxygen and hydrogen atoms in the film could be lost in the form of gases and only carbon atoms are left. With the dose increasing, more and more C-C bonds are cross-linked with the assistance of the so called “knock-on” effect^40^,^41^ of the high-energy e-beam irradiation, which results in the graphitization of the amorphous carbons. The higher e-beam energy, the easier it is to remove oxygen and hydrogen atoms from PMMA and induce cross-link C-C bonds in the residue. The energy of the e-beam used in our experiments is 10 keV and not high enough to directly graphitize the PMMA film well. This is the reason why the negative PMMA is almost insulated before the high-temperature annealing. The high-temperature annealing can further carbonize and graphitize the negative PMMA. Duan *et al.*^40^,^42^ have reported that the PMMA nano-fibers could be directly turned into graphene nano-ribbons by high-energy (200 keV) electron irradiation without high-temperature annealing, which indicates that the annealing is not necessary in preparing the conductive wires (i.e. electrodes) from PMMA if the e-beam energy is high enough.

An interesting and important phenomenon revealed by the HRTEM images is that the graphitic nano-sheets in the annealed PMMA film are “standing” rather than “lying” on the substrate. Since the HRTEM gives a top-down view of the sample, the presence of the layer-by-layer structures with the spacing of about 0.33 nm in the HRTEM images taken at different areas of the cross bar on the Cu grid indicates that the graphite sheets in the negative PMMA are perpendicular to the substrate, which is consistence with the report by Duan *et al.*^35^. The e-beam is perpendicular to the substrate in the EBL and the large e-beam energy is benefit to form the C-C bonds. Therefore, the graphitic sheets tend to align parallel to the direction of the incident e-beam. However, the energy distribution perpendicular to the e-beam is uniform which results in that the sheets extend randomly along the parallel directions of the substrate.

It is convinced that the electrode/graphene contact could be fabricated as the vertical contact between graphite sheet and graphene if the negative PMMA was used as the electrodes in GFET. Based on the analysis above, the microstructure of the all-carbon based GFET can be sketched diagrammatically, as shown in Fig. 2(d). It is reasonable to deduce that the π electrons (i.e. the delocalized electrons) in graphene could be transported easily into the graphite sheets if their π states overlap together. Just like the formation of the C-C bonds in the negative PMMA, the C-C bonds can also be formed among the carbons in the graphene and the negative PMMA by the e-beam irradiation and high-temperature annealing. In this case, the graphene in channel and the graphite-sheet in electrode are bonded together, which makes them form a whole sheet and makes their π states overlap together. Therefore, the vertical contact between the graphene in the channel and the graphite in the electrodes suppliers a quite worth strategy to reduce the impacts of electrode/graphene contacts in GFET.

**Figure 3:**
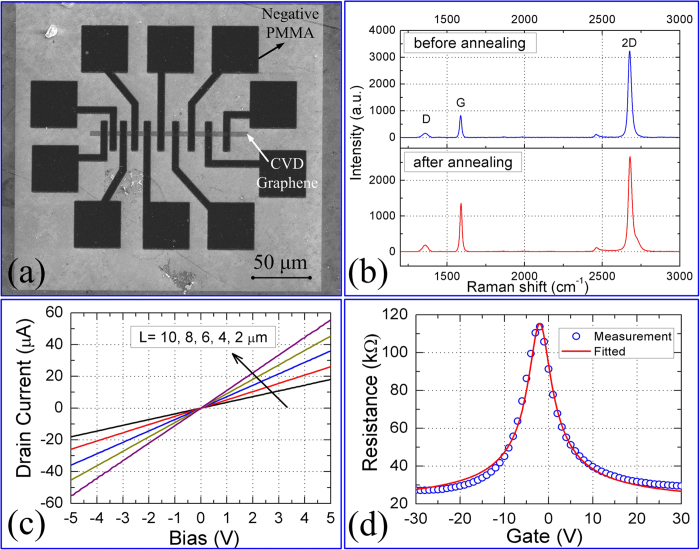
The all-carbon based GFET and its performance. (**a**) The SEM picture of the all-carbon based GFET with different channel lengths from 1 to 10 μm (the scale bar is 50 μm). (**b**) The Raman spectra of the graphene before and after the high-temperature annealing. (**c**) The I–V characteristic of the GFET in (**a**) with the channel length of 2, 4, 6, 8, 10 μm, respectively. (**d**) The transfer characteristic of the GFET with the length of 9 μm. The red line is the theoretical fitting to the measured datum (the blue circles).

Figure 3(a) shows the fabrication process for an all-carbon based GFET with the channel length from 1 to 10 μm. Before the PMMA film for fabricating the electrodes was spin-coated on the substrate, the chemical vapor deposition (CVD) grown graphene^43^ was transferred onto a heavily doping silicon substrate with 300 nm thickness SiO_2_ and etched to a ribbon with the width of 3 μm using O_2_ plasma with the assistant of EBL. The conductive silicon substrate is used as a back-gate of the GFET. From the scanning electron microscope (SEM) image of the Fig. 3(a), it is clear that the electrodes are well defined and the graphene ribbon is well preserved after the high-temperature annealing.

To characterize the graphene in the channel after annealing at the temperature of 800 °C, the Raman spectra of the graphene in the channel before and after the annealing were measured, as shown in Fig. 3(b). It is found that the D peak standing for the defects of the graphene does not enhance obviously, which indicates that the graphene was not damaged in the annealing process. However, the ratio of the 2D to G peaks decreased a little, which may be induced from the tighter adhesion between the substrate and graphene after the high-temperature annealing^44^,^45^. To avoid the damages to the graphene during the annealing process, the slow rising and falling rates of the temperature are necessary to weaken the impact due to the different thermal expansivities of the substrate and graphene.

Any adjacent pair of electrodes in the Fig. 3(a) could be source and drain electrodes, respectively. As shown in Fig. 3(c), the current-voltage (I–V) curves of the GFET with the different channel lengths are standard straight lines, indicating that the electrode/graphene contacts are good and without obvious barriers. The back-gated transfer characteristic curve in Fig. 3(d) also represents that the graphene transistor based on the negative PMMA electrodes is as good as that based on metal electrodes on the field-effect property. In the Fig. 3(d), the blue circles represent the measured datum of the GFET with length of 9 μm and the red solid line represents the fitting by the equation (1) in the Ref [29]. The measurement of the electrical transport properties were performed in the vacuum of ~10^−4^ Pa by a Keithley 4200 analyzer at room temperature, and the carrier mobility of ~3700 cm^2^/V/s of this GFET was obtained from the fitting of the transfer characteristic curve. The Dirac point (the minimum of the transfer characteristic curve) was at about −2 V, indicating that the device was slightly n-type doped. In our previous work^43^, even the best GFET with the Cr/Au electrodes is heavily p-type doped with the Dirac point of about +39 V and its mobility is much smaller than that of this all-carbon based GFET. Moreover, the distinct electron-hole asymmetry (i.e. the asymmetry of the transfer characteristic curve on both sides of the Dirac point) was observed in our previous GFET with the mobility of ~2440 cm^2^/V/s to the electrons and ~1750 cm^2^/V/s to the holes, which is however not so obvious in the all-carbon based GFET as shown in the Fig. 3(d). Therefore, the negative PMMA film composed by the graphite nano-sheets is totally qualified to be the electrode material in GFET.

In summary, we have fabricated a kind of all-carbon based GFET with the carbonaceous electrodes and the channel graphene. The fabrication method of the all-carbon based GFET is simple, convenient and accurate because the electrodes are directly written out by e-beam on a PMMA film. In spite of the simpler and lower cost of the fabrication, the performance of the all-carbon based GFET is totally comparable to the metal contacted GFET from the I–V and field-effect characteristics. It is found that the carbonaceous electrodes are composed by graphite nano-sheets perpendicular to the graphene in the channel. Such vertical contacts between the graphite and graphene supplies a potential choice for the electrode/graphene contact in high-performance GFET.

## Methods

### Carbonaceous electrode preparation

First, a PMMA (MicroChem Corp., 950 K, 2 wt% in chlorobenzene) film was spin-coated on a SiO2/Si substrate followed by 180 °C baking on a hot-plate for 1 minute. Second, the PMMA film was irradiated by a focused e-beam (FEB) in an EBL system (FEI 600i) to define the patterns for electrodes. During the irradiation process, the accelerating voltage and the current of the FEB were 10 kV and 1.4 nA, respectively. In order to transform the PMMA film to a negative resist, the irraidaiton dose should be exceeded 50 times more than the normal dose for a positive PMMA^20^. Third, the irradiated film was cleaned by acetone to dissolve the unirradiated parts of the film so that the patterned negative parts of the PMMA film were left. Final, the patterned sample was annealed at 800 °C for 2 h with the protection of Ar (90% Ar + 10% H_2_ for actual) to improve the conductivity of the negative PMMA parts.

## Additional Information

**How to cite this article**: Chen, W. *et al.* All-carbon based graphene field effect transistor with graphitic electrodes fabricated by e-beam direct writing on PMMA. *Sci. Rep.* **5**, 12198; doi: 10.1038/srep12198 (2015).

## Supplementary Material

Supplementary Information
