# Supplementary material for: All-carbon based graphene field effect transistor with graphitic electrodes fabricated by e-beam direct writing on PMMA
Source: Sci Rep. 2015 Jul 21;5:12198. doi: 10.1038/srep12198 (PMC4508849; doi:10.1038/srep12198)
Supplement: Supplementary Information [file srep12198-s1.pdf]

Supporting Information

All-carbon based graphene field effect transistor with graphitic electrodes fabricated by e-beam direct writing on PMMA

Wei Chen, Yayun Yu, Xiaoming Zheng, Shiqiao Qin, Fei Wang, Jingyue Fang, Guang Wang, Chaocheng Wang, Li Wang, Gang Peng, and Xue-Ao Zhang**

Measurement of the electrical resistivity of the negative PMMA

To measure the electrical resistivity (ρ) of the negative PMMA prepared via the process in the figure 1(a), a series of the wire contacting with two electrodes, as shown in figure S1, were fabricated using different e-beam doses. The length (L) and width (W) of the wire are 100 and 10 μm, respectively. As mentioned in the main text, the e-beam dose is very important to turn the PMMA from positive to negative resist. So it would be reasonable to deduce that the e-beam dose is also a key parameter to determine the electrical property of the negative PMMA. We measured the relationships of the resistance (R) and the thickness (T) to the e-beam dose, respectively, as shown in figure S2. The resistance of the wires was measured by a semiconductor characteristic system (Keithley 4200) at room temperature and the thickness was measured by an atomic force microscope (AFM). One can see that the resistance becomes smaller and smaller with the increase of the e-beam dose and finally tends to be a constant of about 100 kΩ. On the contrary, the thickness increases with the dose increasing and also tends to be constant when the dose is high up to

about 0.3 C/cm^2. The thickness of the negative PMMA after 800 °C annealing at the highest dose was decreased to about 30 nm from the original thickness of 180 nm of the pre-backed PMMA before the e-beam irradiation. The thickness decreasing may come form the following two aspects [19,20]: First, the transformation of the negative PMMA is a cross-linking process, and the cross-linked carbon-carbon covalent bonds may have formed by the e-beam overexposure and high-temperature annealing instead of the van der Waals bonds between macromolecules of PMMA, which results in the film density increased; Second, some volatile groups such as CO_2, H_2, and CH_3O may have formed via electron beam induced decomposition of PMMA .

The resistivity ($\rho = R \cdot W \cdot T / L$) which stands for the conductive ability of this material was calculated under different e-beam doses in figure S3. It is indicated that the resistivity is almost constant with the change of the e-beam dose. And the resistivity of about 4.8×10^{-3} Ω·cm was obtained from the date fitting.

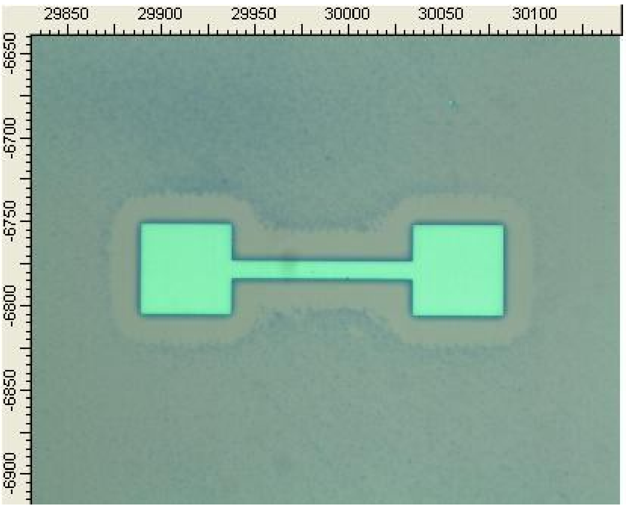

Figure S1 The wire pattern for electrical property measuring

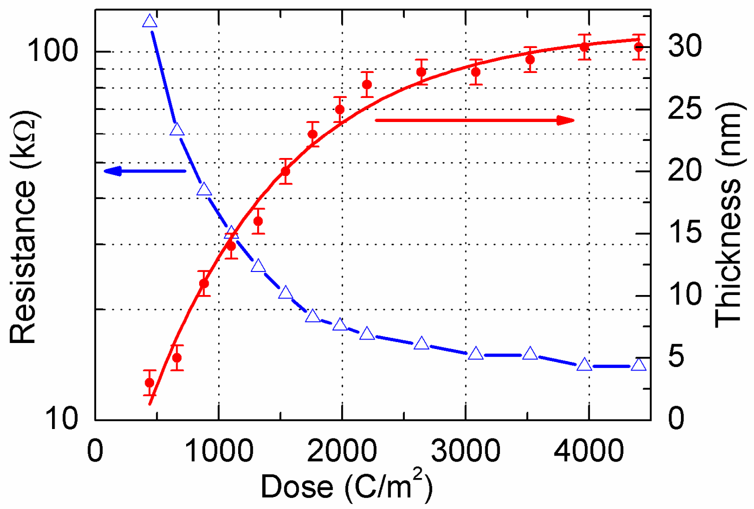

Figure S2 The variations of the Resistance (left) and Thickness (right) with the e-beam dose increasing

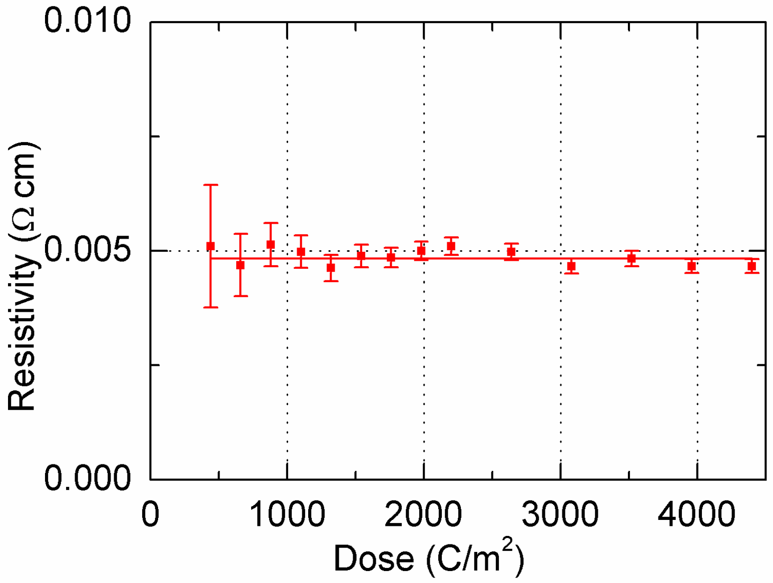

Figure S3 The resistivity of the negative PMMA under different e-beam dose
